# Genetic evidence and multi-omics characterization of DEHP-relevant detoxification and metabolic heterogeneity in hepatocellular carcinoma

**DOI:** 10.3389/ftox.2026.1925431

**Published:** 2026-09-29

**Authors:** Zeyang Fan, Guoqing Zhang, Letian Ren, Wence Zhou

**Affiliations:** 1 The Second Clinical Medical School, Lanzhou University, Lanzhou, China; 2 Department of General Surgery, The Second Hospital of Lanzhou University, Lanzhou, China

**Keywords:** DEHP, detoxification metabolism, hepatocellular carcinoma, molecular subtype, phosphoproteomics, tumor microenvironment

## Abstract

**Background:**

Human evidence linking di(2-ethylhexyl) phthalate (DEHP) exposure to hepatocellular carcinoma (HCC) is limited, and it remains unclear whether DEHP-relevant detoxification biology captures clinically meaningful HCC heterogeneity.

**Methods:**

We integrated two-sample Mendelian randomization, proteomic and phosphoproteomic subtyping, bulk, single-cell and spatial transcriptomics, computational candidate prioritization, molecular docking, and cell-based assays. Genetically proxied MECPP and MEHHP were evaluated for associations with HCC and liver-related outcomes. DPTS-related molecular, cellular, spatial, and experimental patterns were then characterized.

**Results:**

MR did not support a causal association of genetically proxied MECPP or MEHHP with HCC. In 165 CPTAC tumors, consensus clustering identified two detoxification-phosphorylation tumor subtypes. Compared with DPTS1, DPTS2 showed coordinated suppression of xenobiotic metabolism, bile acid metabolism, fatty-acid oxidation, phase II conjugation, and transport programs. A transcriptomically projected DPTS2-like state was associated with poorer overall survival in TCGA-LIHC, but this association was not replicated in ICGC-LIRI-JP. Single-cell and spatial analyses localized these programs mainly to hepatocyte-like compartments and showed modest spatial coordination with nine prioritized genes, including ALDH2, EHHADH, and PCK2. LINCS-based screening and molecular docking prioritized PD-0325901, trametinib, and regorafenib, but these predictions did not demonstrate binding or efficacy, and the compounds were not tested experimentally. Basal qPCR and DEHP-treatment experiments showed cell-line-specific expression and phenotypic responses.

**Conclusion:**

DPTS defines a DEHP-relevant, detoxification-suppressed HCC molecular phenotype rather than an exposure-defined or DEHP-induced subtype. These findings provide a bounded framework for future mechanistic validation of metabolic vulnerability in HCC.

## 1 Introduction

Hepatocellular carcinoma (HCC) remains a major contributor to cancer mortality within a changing global cancer burden. The American Cancer Society projected approximately 2,114,850 new cancer cases and 626,140 cancer deaths in the United States in 2026. The same report showed that the 5-year relative survival rate for liver cancer increased from 7% in the mid-1990s to 22% for diagnoses made during 2015–2021, although liver cancer remains a high-mortality malignancy (Siegel et al., 2026). Globally, HCC continues to represent a leading cause of cancer-related death, while its etiological distribution is shifting as the contribution of metabolic dysfunction-associated steatotic liver disease and alcohol-related liver disease increases relative to viral hepatitis (Toh et al., 2023; Hwang et al., 2025). In addition to its etiological diversity, HCC exhibits substantial molecular, metabolic, and microenvironmental heterogeneity, which contributes to differences in disease progression, therapeutic response, and clinical outcome (Xing et al., 2023; Park and Hall, 2025).

Di(2-ethylhexyl) phthalate (DEHP) is a widely used plasticizer, and human exposure is commonly assessed using urinary oxidative metabolites such as mono-(2-ethyl-5-carboxypentyl) phthalate (MECPP) and mono-(2-ethyl-5-hydroxyhexyl) phthalate (MEHHP). Recent population-based studies have reported associations between urinary DEHP metabolite burden and non-alcoholic fatty liver disease or metabolic dysfunction-associated steatotic liver disease (He et al., 2023; Zou et al., 2024). However, the available human studies are predominantly cross-sectional and therefore cannot establish temporal or causal relationships between phthalate exposure and liver disease (Gogola et al., 2025). Authoritative toxicological evaluations classify DEHP as reasonably anticipated to be a human carcinogen based on sufficient evidence of carcinogenicity in experimental animals (National Toxicology Program, 2021a). In the NTP Technical Report 601, dietary DEHP exposure produced clear evidence of carcinogenic activity in both male and female Sprague Dawley rats, based partly on increased incidences of hepatocellular adenoma or carcinoma (National Toxicology Program, 2021b). However, the NTP evaluation also concluded that the available epidemiological data were inadequate to determine the relationship between human cancer and exposure specifically to DEHP (National Toxicology Program, 2021a). Consistently, a recent review concluded that the specific contribution of DEHP to cancer initiation and progression in humans remains insufficiently clarified (Zhou et al., 2025). Mechanistic evaluations have further identified substantial species differences in DEHP metabolism, PPARA activation, hepatocyte proliferation, and other pathways involved in rodent hepatocarcinogenesis, which complicate direct extrapolation of rodent liver tumors to human HCC (Rusyn et al., 2006; Rusyn and Corton, 2012; Ito et al., 2014). Therefore, the available evidence supports DEHP-associated hepatocarcinogenicity in experimental animals but does not establish that DEHP causes HCC in humans.

Against this evidence hierarchy, the purpose of the downstream analyses was not to infer individual DEHP exposure or to establish a DEHP-driven carcinogenic mechanism, but to determine whether detoxification and metabolic processes relevant to DEHP-responsive liver biology could characterize molecular heterogeneity within established HCC. The absence of established human evidence for DEHP-induced HCC does not necessarily exclude the biological relevance of DEHP-responsive hepatic pathways in established liver cancer. DEHP-related hepatic effects converge on lipid metabolism, redox regulation, aldehyde handling, xenobiotic metabolism, and inflammatory responses, many of which are also altered during HCC progression (Park and Hall, 2025; Huang et al., 2022). Accordingly, DEHP may be more appropriately investigated as a potential modifier of hepatic metabolic and detoxification states than as a confirmed initiating carcinogen in human HCC. Under this framework, molecular features associated with DEHP-responsive detoxification biology can be used to examine HCC heterogeneity, provided that they are not interpreted as direct measurements of individual exposure or as evidence of a DEHP-specific tumor mechanism.

Molecular classifications have demonstrated that HCC comprises biologically distinct subgroups characterized by differences in metabolic activity, kinase signaling, immune architecture, prognosis, and therapeutic sensitivity. Integrated genomic, transcriptomic, proteomic, and phosphoproteomic analyses have further shown that protein- and phosphorylation-level information can identify clinically and biologically relevant HCC subtypes that are not fully captured by transcriptomic measurements alone (Xing et al., 2023; Li B. et al., 2024). These findings provide a rationale for integrating proteomic and phosphoproteomic features related to hepatic detoxification and metabolism when characterizing HCC molecular states. Importantly, such a classification describes tumor-associated molecular heterogeneity and does not, in the absence of exposure data, identify the environmental cause of that heterogeneity.

Single-cell and spatially resolved analyses have further demonstrated that HCC contains heterogeneous malignant, hepatocyte-like, immune, stromal, and endothelial populations whose interactions vary across patients and tissue regions (Lu et al., 2022). Spatial profiling has identified immunosuppressive macrophage states and regional immune architectures associated with HCC progression and therapeutic response (Qiu et al., 2024). Spatial transcriptomic studies have also revealed distinct tissue programs associated with treatment resistance and recurrence (Zhang et al., 2023). These observations indicate that detoxification and metabolic programs identified in bulk tumors should be evaluated in their cellular and spatial contexts, while recognizing that marker-based localization alone cannot establish cellular lineage or causal interactions.

Here, we integrated Mendelian randomization, proteomic and phosphoproteomic subtyping, external bulk-transcriptomic projection, single-cell and spatial transcriptomic analyses, pathway-level evidence integration, computational candidate prioritization, molecular docking, and cell-based assays. We first evaluated whether genetically proxied DEHP metabolites were associated with HCC or other liver-related outcomes. Because neither the genetic analyses nor the tumor cohorts provided evidence of individual DEHP exposure, we subsequently investigated whether a DEHP-relevant detoxification and metabolic feature set could define reproducible molecular heterogeneity within HCC. The resulting detoxification-phosphorylation tumor subtype (DPTS) was therefore interpreted as a DEHP-relevant molecular phenotype rather than a DEHP-induced or exposure-defined HCC subtype. We further examined its cross-cohort consistency, cellular and spatial distribution, candidate molecular features, and experimentally testable associations while maintaining a distinction between computational prioritization and mechanistic validation.

## 2 Methods

### 2.1 Study design and Mendelian randomization analysis

This study integrated genetic proxy analysis, tumor multi-omics characterization, and *in vitro* experiments to investigate DEHP-relevant detoxification and metabolic alterations in hepatocellular carcinoma (HCC). Two-sample Mendelian randomization (MR) was performed using genome-wide association study summary statistics for the DEHP metabolites MECPP and MEHHP as exposures. The outcomes included non-alcoholic fatty liver disease, liver fibrosis or cirrhosis, toxic liver disease, and HCC from FinnGen release 12, together with ALT, AST, ALP, GGT, and total bilirubin from the UK Biobank Neale Lab resource. Four variants, rs1359232, rs117529685, rs117033379, and rs4918798, were retained for MECPP, whereas rs7895726 was retained for MEHHP. Effect alleles were harmonized before analysis, and F statistics below 10 were considered indicative of potential weak-instrument bias. First-order fixed-effect inverse-variance weighting was used for MECPP, whereas the Wald ratio was used for the single-variant MEHHP instrument. MECPP sensitivity analyses included Cochran’s Q test, weighted-median estimation with 5,000 bootstrap replicates, MR-Egger regression, and leave-one-out analysis (Bowden et al., 2015; Bowden et al., 2016). MR-Egger analysis was considered exploratory because only four MECPP instruments were available, while sensitivity analyses were not estimable for MEHHP. Because the original linkage disequilibrium clumping records were unavailable, variant independence could not be independently verified. Individual-level DEHP exposure was not measured in the tumor cohorts; therefore, the downstream DPTS was interpreted as a DEHP-relevant molecular phenotype rather than an exposure-defined HCC subtype. The reporting of the MR analysis was guided by the STROBE-MR recommendations (Skrivankova et al., 2021).

### 2.2 Proteomic and phosphoproteomic identification of DPTS subtypes

Proteomic and phosphoproteomic data from 165 CPTAC HCC tumors with matched measurements were used to identify DPTS subtypes. A curated panel of 168 genes covering cytochrome P450 metabolism, phase II conjugation, redox and aldehyde metabolism, transport, bile and lipid metabolism, xenobiotic stress regulation, and MR-related candidates was matched to 144 protein and 85 phosphosite measurements. The resulting 229 features were standardized across samples and integrated using the ConsensusClusterPlus implementation of consensus clustering (Wilkerson and Hayes, 2010). Candidate cluster numbers from K = 2 to K = 6 were evaluated using 100 resampling iterations, with 80% of samples and 80% of features included in each iteration. K-means clustering was applied within each resample, and final assignments were obtained by average-linkage hierarchical clustering of the consensus matrix. The optimal cluster number was selected according to the proportion of ambiguous clustering and mean silhouette width (Șenbabaoğlu et al., 2014). The cluster with the higher mean detoxification feature score was designated DPTS1, whereas the other cluster was designated DPTS2. Principal component analysis was used to visualize subtype separation. Differences in individual protein and phosphosite features were evaluated using Welch’s t-test followed by Benjamini-Hochberg correction. Subtype robustness was further examined through 300 independent feature-resampling analyses, each using 184 of the 229 features and Ward.D2 hierarchical clustering with K = 2. Agreement with the original assignments was assessed using the adjusted Rand index, sample-level consensus, and within-subtype versus between-subtype pairwise consensus.

### 2.3 Multi-evidence candidate prioritization and external projection of DPTS

Candidate genes were prioritized by integrating their detection in the CPTAC proteome and phosphoproteome, DPTS-associated feature status, magnitude and false discovery rate of the DPTS2 versus DPTS1 difference, inclusion in the external transcriptomic signature, and expression in hepatocyte-like cells. The heuristic priority score assigned 2.0 points for DPTS marker status, 1.5 points for protein detection, 1.0 point for phosphosite detection, and 1.0 point for inclusion in the external signature. Additional contributions were derived from the absolute DPTS difference, capped at 2.0 points; marker significance, calculated from the negative log10 false discovery rate and capped at 2.0 points; and the percentage of hepatocyte-like cells expressing the gene, capped at 2.0 points. An investigator-defined bridge-priority term ranging from 0 to 5 points was included to favor candidates prespecified for downstream biological assessment. Because this heuristic term could alter the rank order, the resulting list was treated as a hypothesis-generating candidate set rather than an objective or statistically derived ranking. For external projection, a transcriptomic signature was constructed from the 50 strongest DPTS1-associated genes and four DPTS2-relative-up genes. Gene expression was standardized within each external cohort, and a DPTS suppression score was calculated by contrasting the mean expression of DPTS2-relative-up genes with that of DPTS1-associated genes. Within each source dataset, the suppression-score cutoff was defined as the median calculated from the complete expression matrix before restriction to HCC samples with available survival data and patient-level deduplication. This fixed cutoff was then applied to the final survival-analysis subset. Therefore, the resulting DPTS1-like and DPTS2-like groups were not expected to be equal in size. Overall survival was evaluated in TCGA-LIHC and ICGC-LIRI-JP using Kaplan-Meier and Cox regression analyses. TCGA models were adjusted for age, sex, and tumor stage, with vascular invasion and time-varying effects examined in sensitivity analyses. ICGC estimates were analyzed without multivariable adjustment because of the limited number of events and incomplete covariate information. Because DPTS was originally defined using CPTAC proteomic and phosphoproteomic features, its application to TCGA-LIHC and ICGC-LIRI-JP represented transcriptome-based cross-platform projection. These analyses assessed whether a related transcriptomic state was detectable in external cohorts and did not constitute independent proteomic or phosphoproteomic validation of the original DPTS classification.

### 2.4 Single-cell transcriptomic localization and sample-aware pathway analysis

Single-cell RNA sequencing data from GSE290925, comprising 12 HCC samples, were used to determine the cellular distribution of the DPTS program. A 19-gene DPTS1 detoxification module containing *PCK2, EHHADH, SLC27A5, OTC, HADH, HSD17B6, GLYAT, MLYCD, CYP8B1, ACOX2, ECHDC2, SCP2, ALDH4A1, ALDH2, SULT1A1, SULT1A2, GSTK1, ABCC6, and UGT2B4* was scored as the mean log-normalized expression of the genes detected in each cell. Cell-type identities were inferred by comparing marker-module scores for hepatocyte-like, T or NK, myeloid, B-cell, endothelial, and fibroblast populations; cells without a positive marker-module score were classified as unclassified. These assignments were considered marker-based inferred identities rather than reference-mapped annotations. For UMAP visualization, cells with at least 200 detected genes, at least 500 RNA counts, and no more than 25% mitochondrial transcripts were retained, with random downsampling to a maximum of 60,000 cells. Candidate-gene expression was summarized by inferred cell type. To avoid cell-level pseudo-replication, DPTS scores were averaged for each sample and cell type, and hepatocyte-like values were compared with other cell types across 12 paired samples using paired Wilcoxon tests. Hepatocyte-like cells were also divided at the median DPTS score into DPTS-high and DPTS-low states. For pathway analysis, raw counts from these states were aggregated within each biological sample, and an edgeR quasi-likelihood model including sample and DPTS state was used to generate gene-level statistics (Robinson et al., 2010). Reactome enrichment was evaluated for six prespecified detoxification and metabolic pathways, and sample-specific enrichment directions were calculated separately (Milacic et al., 2024).

### 2.5 Spatial transcriptomic analysis of DPTS-associated programs

Exploratory spatial transcriptomic analysis was performed using two HCC tumor sections from GSE245908, CHC20 (GSM7850822) and CHC23 (GSM7850823). According to the GEO records, both specimens were OCT-embedded 10-μm HCC sections annotated as receiving no treatment and were processed using the 10x Genomics Visium platform; tissues with RNA integrity numbers of at least 6.8 were included. Sample-specific age, sex, etiology, tumor stage, and DPTS status were unavailable. The two specimens were therefore analyzed independently as exploratory HCC sections rather than as representatives of predefined DPTS subtypes (Giraud et al., 2024). Only in-tissue spots were retained. Counts were normalized to 10,000 transcripts per spot and log1p transformed, and data quality was assessed using total counts, detected features, and mitochondrial transcript percentage. Spot-level scores were calculated as the mean log-normalized expression of the available genes in each predefined module. To prevent mathematical overlap, the no-core DPTS score was calculated from the 19-gene DPTS detoxification module after excluding *PCK2, ALDH2, ACOX2, SLC27A5, SULT1A1, GSTK1, EHHADH, SCP2*, and *ABCC6*; these nine genes were used separately to calculate the core-target score. Immune and stromal scores were derived from myeloid, T/NK-cell, B-cell, fibroblast, and endothelial modules. Candidate-derived proxy scores were calculated for fatty-acid oxidation, peroxisomal metabolism, PPARA-related metabolism, bile-acid metabolism, and xenobiotic metabolism. Associations were evaluated within each specimen using Spearman correlation. Partial Spearman correlations were obtained by rank transforming the variables and correlating their residuals after adjustment for the hepatocyte-like score, nCount_Spatial, and nFeature_Spatial; the hepatocyte-like association was adjusted for the two technical variables only. Because only two spatial specimens were available, neighboring spots were not statistically independent, and neither specimen had an independently assigned DPTS status, all spatial correlations and spot-level P values were considered exploratory and were not interpreted as subtype-level validation.

### 2.6 Cross-cohort pathway scoring and exploratory bulk axis-score analysis

To evaluate whether DPTS-associated transcriptomic differences were reproduced across external HCC cohorts, we restricted the TCGA-LIHC and ICGC-LIRI-JP validation analyses to samples annotated as HCC and classified as DPTS1-like or DPTS2-like. Gene expression values were z-transformed by gene within each cohort, and predefined detoxification, xenobiotic metabolism, bile acid metabolism, lipid metabolism, oxidative stress, proliferation, immune and stromal modules were summarized as mean z-score signatures across matched genes. Differences between DPTS1-like and DPTS2-like tumors were assessed using Wilcoxon rank-sum tests, followed by Benjamini-Hochberg false-discovery-rate correction. Preranked gene set enrichment analysis was performed using gene-level differential statistics contrasting DPTS2-like with DPTS1-like tumors, with Reactome and curated custom gene sets used to characterize pathway-level changes (Milacic et al., 2024; Subramanian et al., 2005). To compare bulk and single-cell evidence, selected Reactome enrichment results from hepatocyte-like pseudobulk profiles were aligned with bulk cohort enrichment patterns. For exploratory crosstalk-associated analyses, predefined ligand-receptor axis gene sets were converted into bulk sample-level axis scores using the same z-score averaging strategy, and their associations with pathway/module scores were assessed using Spearman correlation. These analyses were interpreted as transcriptomic pathway and bulk axis-score associations, rather than as direct evidence of ligand-receptor activity, cell-cell communication, DEHP exposure, or independent protein/phosphoprotein validation.

### 2.7 Computational drug-reversal prioritization, molecular docking, and experimental characterization

Drug-reversal candidates were prioritized from LINCS HepG2 perturbation signatures according to their ability to counteract the DPTS2/high-risk expression pattern, together with cell-line evidence and structural availability. The top three compounds, PD-0325901, Trametinib, and Regorafenib, were evaluated against ALDH2, PCK2, and EHHADH. The ALDH2 receptor was derived from the human ALDH2 crystal structure 1O02, whereas AlphaFold models Q16822 v6 and Q08426 v6 were used for PCK2 and EHHADH, respectively. Ligand structures were obtained from PubChem and prepared together with the receptor structures for docking using AutoDock Vina 1.2.7 (Trott et al., 2010; Eberhardt et al., 2021). The docking exhaustiveness was 32, with 10 output modes, an energy range of 3 kcal/mol, and a fixed random seed of 20260618. For experimental characterization, CCK-8 assays were performed at 0, 12, 24, and 48 h across vehicle and 25, 50, 100, and 200 μM DEHP conditions. Basal ALDH2, EHHADH, and PCK2 mRNA levels were measured in LO2, Huh7, and Hep-3B cells using the 2^^−ΔΔCt^ method, with qPCR statistics performed on ΔCt values and Holm correction. Western blotting was performed only for ALDH2 and EHHADH under vehicle, 10 μM, and 25 μM conditions. Transwell migration and EdU incorporation were assessed in Huh7 and Hep-3B cells under vehicle and 25 μM DEHP, using three image fields per condition for image-based quantification. Docking and cell-based experiments were interpreted as complementary, hypothesis-generating evidence; the docking compounds were not directly tested for pharmacological efficacy in these experiments.

## 3 Results

### 3.1 Genetic associations of DEHP metabolite proxies with liver-related outcomes

The integrated study design and its evidence boundaries are summarized in Figure 1, and the MR workflow is shown in Figure 2A. The four MECPP instruments had F statistics of 9, 121, 144, and 81, whereas the single MEHHP instrument had an F statistic of 9, indicating potential weak-instrument bias for one MECPP variant and the MEHHP variant (Figure 2B; Supplementary Figure S1C; Supplementary Table S1). Neither MECPP nor MEHHP was associated with HCC: the MECPP estimate was beta = 0.230, SE = 0.496, and P = 0.643, whereas the MEHHP estimate was beta = 0.678, SE = 2.001, and P = 0.735. The MECPP-HCC association showed no substantial heterogeneity or directional pleiotropy, and all leave-one-out estimates remained non-significant. Neither metabolite proxy was associated with non-alcoholic fatty liver disease, fibrosis or cirrhosis, or toxic liver disease (Figure 2C; Supplementary Figure S1A). For biochemical traits, MECPP was inversely associated with AST (beta = −0.077, SE = 0.024, P = 0.00162) and positively associated with ALP (beta = 0.088, SE = 0.025, P = 4.02 × 10^^−4^), while the single-variant MEHHP proxy was positively associated with ALP (beta = 0.404, SE = 0.097, P = 2.91 × 10^^−5^; Figure 2D; Supplementary Figure S1B). However, the MECPP-ALP estimate showed substantial heterogeneity and an MR-Egger intercept signal, whereas the MEHHP-ALP result relied on one potentially weak instrument. These biochemical associations were therefore considered exploratory. Complete SNP-level estimates, method comparisons, heterogeneity assessments, and leave-one-out analyses are provided in Supplementary Figures S1D–G and Supplementary Table S1. Overall, the MR results did not establish genetically proxied MECPP or MEHHP as causal determinants of HCC. The subsequent analyses therefore evaluated DPTS-related detoxification and metabolic programs as associative features of HCC heterogeneity rather than as an exposure-defined causal subtype.

**FIGURE 1:**
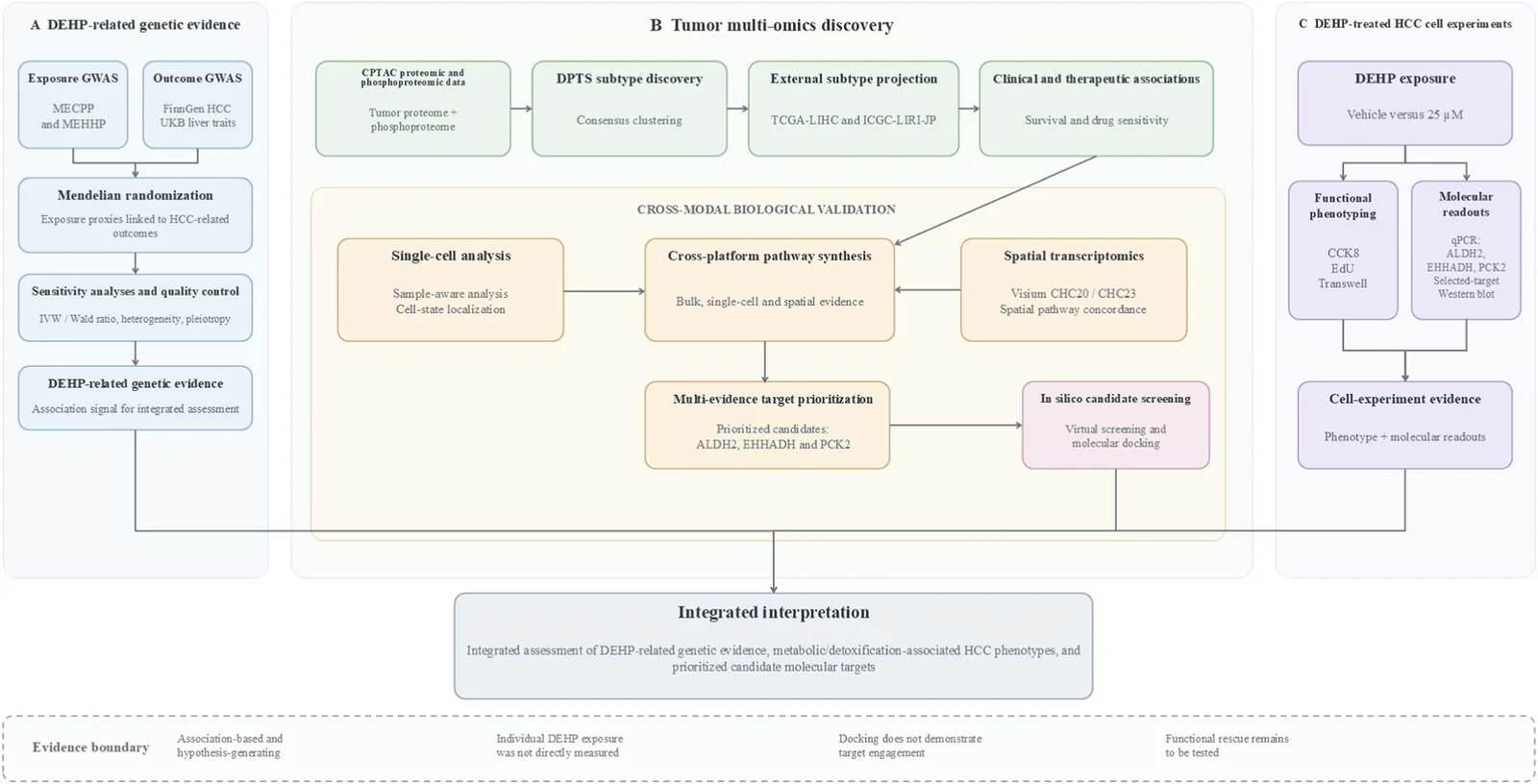
Integrated framework for investigating DEHP-relevant alterations in HCC. **(A)** Discovery and model construction, including data resources, integrated multi-omics analysis, feature selection, clinical filtering, and the six-gene model. **(B)** Mechanistic characterization, including single-cell analysis, spatial transcriptomics, functional interpretation, and immune/mutation context. **(C)** Experimental validation and conclusion, including qPCR validation and the main conclusion.

**FIGURE 2:**
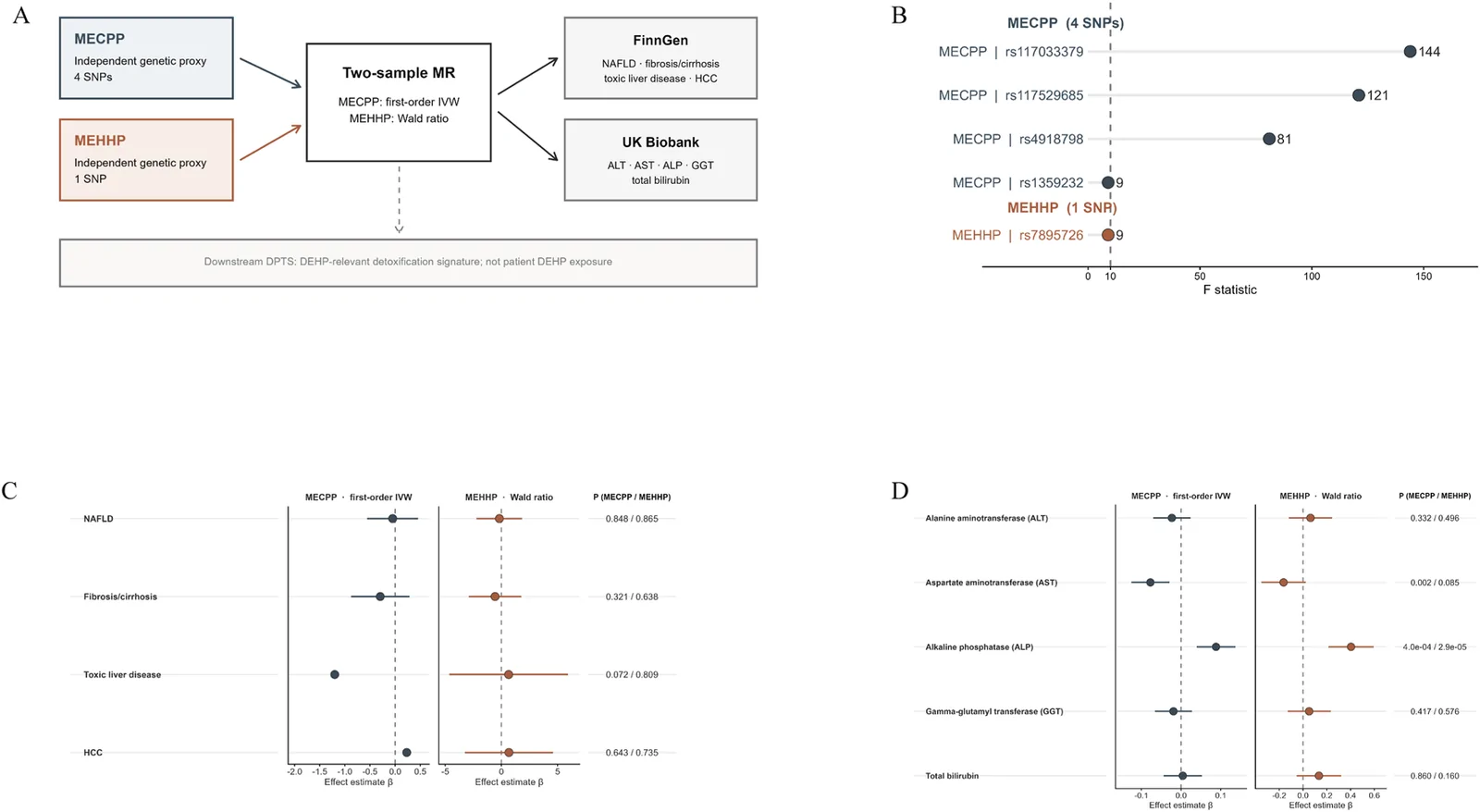
Mendelian randomization analysis of DEHP metabolite proxies and liver-related outcomes. **(A)** MR workflow. **(B)** Instrument F statistics. **(C)** Associations with FinnGen liver disease outcomes. **(D)** Associations with UK Biobank biochemical traits. MECPP estimates were obtained using first-order fixed-effect inverse-variance weighting, whereas MEHHP estimates were obtained using the Wald ratio. Points and lines indicate beta estimates and 95% confidence intervals.

### 3.2 Identification and robustness assessment of two DPTS subtypes

Among the candidate solutions, K = 2 had the lowest proportion of ambiguous clustering value of 0.086 and the highest mean silhouette width of 0.178, supporting selection of a two-subtype solution (Figure 3A; Supplementary Figures S2D,E). The consensus matrix and principal component analysis showed separation between the two groups, with the first two principal components explaining 33.0% and 7.8% of the variation, respectively (Figures 3B,C). The resulting classification included 86 DPTS1 tumors and 79 DPTS2 tumors (Figure 3D). DPTS1 had a higher mean detoxification feature score than DPTS2 (0.391 versus −0.425), supporting their descriptive designation as detoxification-active and detoxification-suppressed states, respectively (Figure 3E). Of the 229 unique input features, 205 differed between the subtypes at FDR < 0.05, including 200 protein or phosphosite features that were higher in DPTS1 and five that were higher in DPTS2. The DPTS1-associated pattern included proteins involved in bile and lipid metabolism, aldehyde handling, xenobiotic metabolism, and transport, with PCK2, EHHADH, and SLC27A5 among the most strongly differentiated protein features (Figure 3F; Supplementary Figures S2A–C). In the 300 feature-resampling analyses, the median adjusted Rand index relative to the original classification was 0.500, with an interquartile range of 0.386–0.648 (Figure 3G). Mean pairwise consensus was higher within the original subtypes than between them (0.800 versus 0.298), indicating reproducible group-level structure despite moderate sample-level reassignment under feature perturbation (Figure 3H; Supplementary Figures S2F,G). These findings support DPTS as a reproducible but not invariant detoxification and metabolic classification. Because patient-level DEHP exposure was unavailable, the subtypes represent DEHP-relevant molecular states rather than confirmed DEHP-induced tumor states.

**FIGURE 3:**
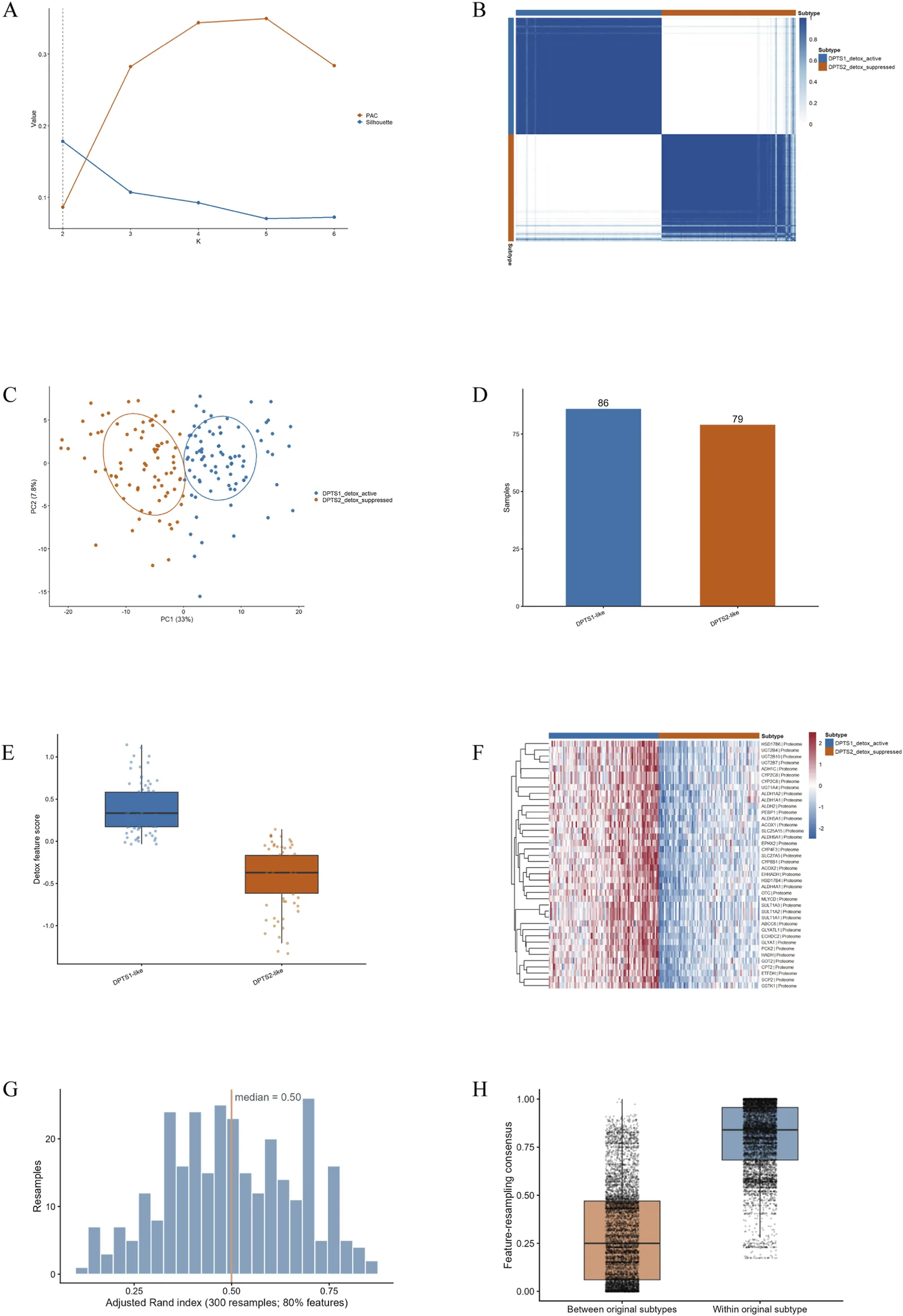
Proteomic and phosphoproteomic identification of DPTS subtypes in CPTAC HCC. **(A)** Selection of the cluster number according to the proportion of ambiguous clustering. **(B)** Consensus matrix for the selected K = 2 solution. **(C)** Principal component analysis of the integrated protein and phosphosite features. **(D)** Numbers of tumors assigned to DPTS1 and DPTS2. **(E)** Integrated detoxification feature scores in the two subtypes. **(F)** Heatmap of the 25 most strongly differentiated protein or phosphosite features ranked by false discovery rate. **(G)** Distribution of adjusted Rand index values from 300 feature-resampling analyses. The vertical line indicates the median value. **(H)** Pairwise feature-resampling consensus within and between the original DPTS subtypes. DPTS1 and DPTS2 denote detoxification-active and detoxification-suppressed molecular states, respectively, and do not represent measured DEHP exposure groups.

### 3.3 Multi-evidence prioritization identifies candidate genes associated with DPTS and clinical outcome

Integration of protein, phosphosite, DPTS-marker, external-transcriptomic, and single-cell evidence generated a heuristic ranking of candidate genes (Figures 4A,B). Nine genes were prioritized for downstream assessment: PCK2, ALDH2, ACOX2, SLC27A5, SULT1A1, GSTK1, SCP2, EHHADH, and ABCC6 (Figure 4C; Supplementary Figures S3C–E). These genes represent a hypothesis-generating candidate set rather than objectively identified core targets. Most prioritized proteins were lower in DPTS2 than in DPTS1, consistent with the detoxification-suppressed phenotype, although phosphosite coverage varied among candidates (Figures 4D,H; Supplementary Figures S3A,B). External projection classified 195 of 341 TCGA-LIHC tumors as DPTS2-like and 146 as DPTS1-like. DPTS2-like tumors had poorer overall survival in the unadjusted analysis (hazard ratio = 1.76, 95% CI, 1.21 to 2.57; P = 0.0034), and the association remained after adjustment for age, sex, and stage (hazard ratio = 1.78, 95% CI, 1.18 to 2.71; P = 0.0066; Figures 4E,F). The continuous DPTS suppression score was also associated with increased mortality risk in TCGA-LIHC (hazard ratio per standard deviation = 1.27, 95% CI, 1.09 to 1.48; P = 0.0023). However, the vascular-invasion-adjusted sensitivity model included only 95 patients and did not retain statistical support. The unadjusted proportional hazards test indicated possible time dependence, whereas the age-, sex-, and stage-adjusted model showed no detectable violation and no significant log-time interaction (P = 0.461; Supplementary Figures S3F,G). In ICGC-LIRI-JP, 133 of 163 tumors were classified as DPTS2-like and only 30 as DPTS1-like, with 28 deaths overall. The survival association was not replicated in this cohort (hazard ratio = 1.25, 95% CI, 0.43 to 3.60; P = 0.681; Figure 4G; Supplementary Figures S3F–H). DPTS showed an adverse prognostic association in TCGA-LIHC, but the lack of replication and sparse ICGC events require cautious interpretation. The external analyses supported the clinical and pathway relevance of a projected transcriptomic state but did not independently validate the CPTAC-derived DPTS classification at the protein or phosphosite level. The candidate ranking and survival findings should therefore be regarded as hypothesis-generating rather than as evidence of DEHP-specific targets or independently validated prognostic biomarkers.

**FIGURE 4:**
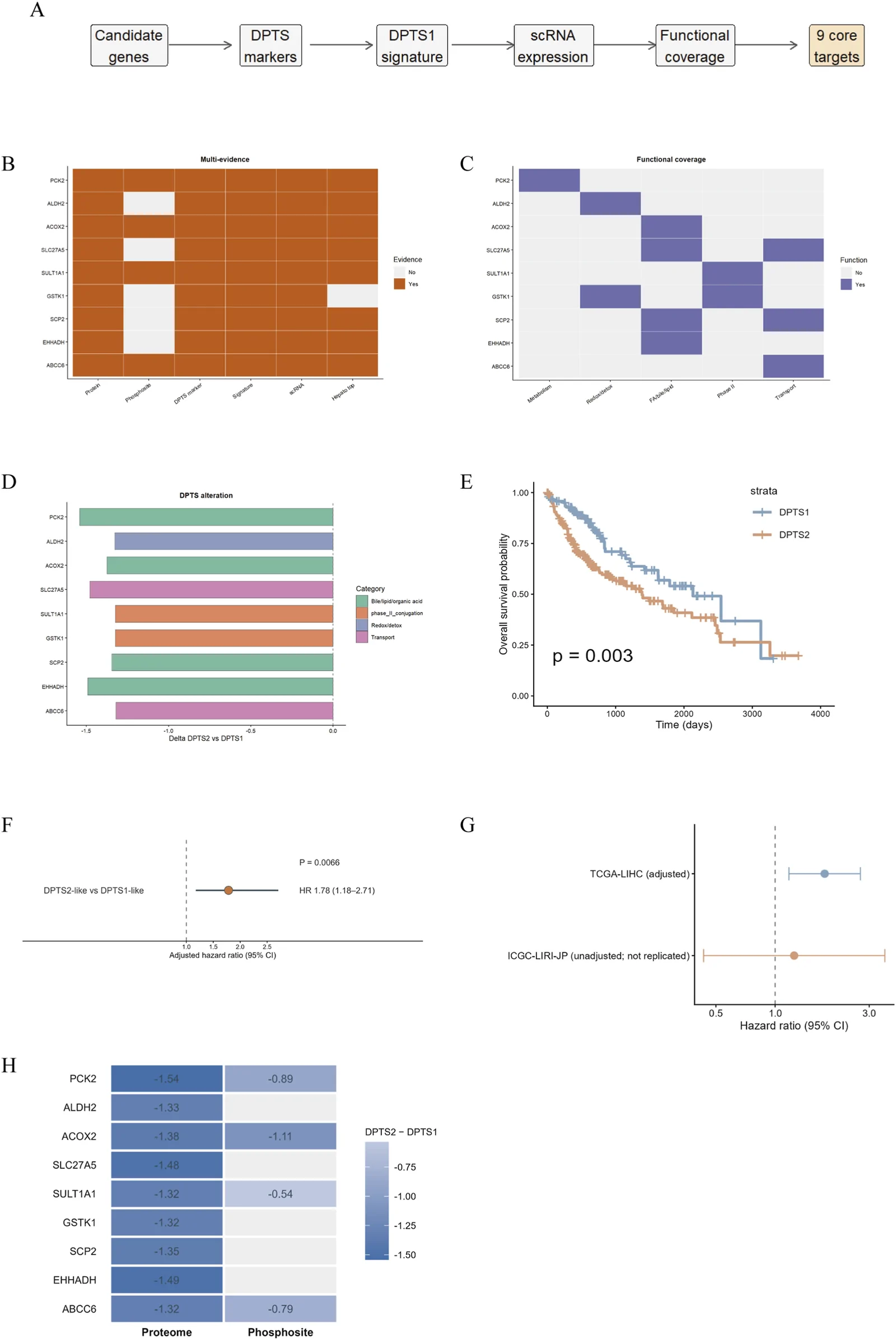
Multi-evidence candidate prioritization and external clinical projection of DPTS. **(A)** Workflow for integrating molecular evidence and prioritizing candidate genes. **(B)** Evidence matrix for the nine highest-ranked candidates across transcriptomic, proteomic, phosphosite, DPTS, single-cell, and spatial datasets. **(C)** Functional categories represented by the prioritized candidates. **(D)** Protein-level differences between DPTS2 and DPTS1 for the prioritized genes. **(E)** Kaplan-Meier analysis of overall survival according to projected DPTS group in TCGA-LIHC. **(F)** Cox regression estimate for DPTS2-like versus DPTS1-like tumors after adjustment for age, sex, and stage in TCGA-LIHC. **(G)** Comparison of survival effect estimates between TCGA-LIHC and ICGC-LIRI-JP. The ICGC estimate was unadjusted and did not replicate the TCGA association. **(H)** Protein and phosphosite differences between DPTS2 and DPTS1 for the prioritized candidates. Negative values indicate lower abundance in DPTS2. TCGA-LIHC and ICGC-LIRI-JP represent transcriptome-based cross-platform projections of the CPTAC-derived DPTS classification and do not constitute independent proteomic or phosphoproteomic validation.

### 3.4 The DPTS program is preferentially localized to hepatocyte-like cells

UMAP visualization identified hepatocyte-like, immune, stromal, and endothelial cell populations across the GSE290925 dataset (Figure 5A), while candidate-gene analysis showed that ALDH2, EHHADH, PCK2, and other DPTS-associated genes were detectable in the hepatocyte-like compartment (Figure 5B; Supplementary Figure S4C). The DPTS detoxification score was distributed across several populations but was most prominent in hepatocyte-like cells (Figure 5C). At the biological-sample level, hepatocyte-like cells had the highest mean DPTS score across cell types, with a mean of 0.182 compared with 0.033–0.097 in the other inferred populations (Figure 5D; Supplementary Figures S4D,E). Hepatocyte-like cells ranked first within 10 of the 12 samples, although they ranked third in CJ1 and fifth in YHL0 (Figure 5E; Supplementary Figure S4F). Paired sample-level comparisons supported higher hepatocyte-like DPTS scores relative to T or NK, myeloid, fibroblast, B-cell, endothelial, and unclassified populations, with P values ranging from 0.0025 to 0.0054. Median division of hepatocyte-like cells produced descriptive DPTS-high and DPTS-low states (Figure 5F). Sample-adjusted pseudobulk analysis showed positive enrichment of all six prespecified pathways in the DPTS-high state, including peroxisomal lipid metabolism, bile acid metabolism, PPARA-regulated gene expression, mitochondrial fatty-acid beta-oxidation, glutathione conjugation, and xenobiotic metabolism, with normalized enrichment scores from 1.50 to 1.89 and false discovery rates of 0.0164 or lower (Figure 5G). The positive enrichment direction was maintained across the evaluable biological samples, although the strength and statistical support varied among samples and pathways (Figure 5H). Sample-level quality and cell-composition summaries are provided in Supplementary Figures S4A,B. These findings localize the DPTS program predominantly to hepatocyte-like cells and support its association with hepatic detoxification and metabolic functions, but they do not demonstrate DEHP exposure, lineage specificity, or a causal cell state.

**FIGURE 5:**
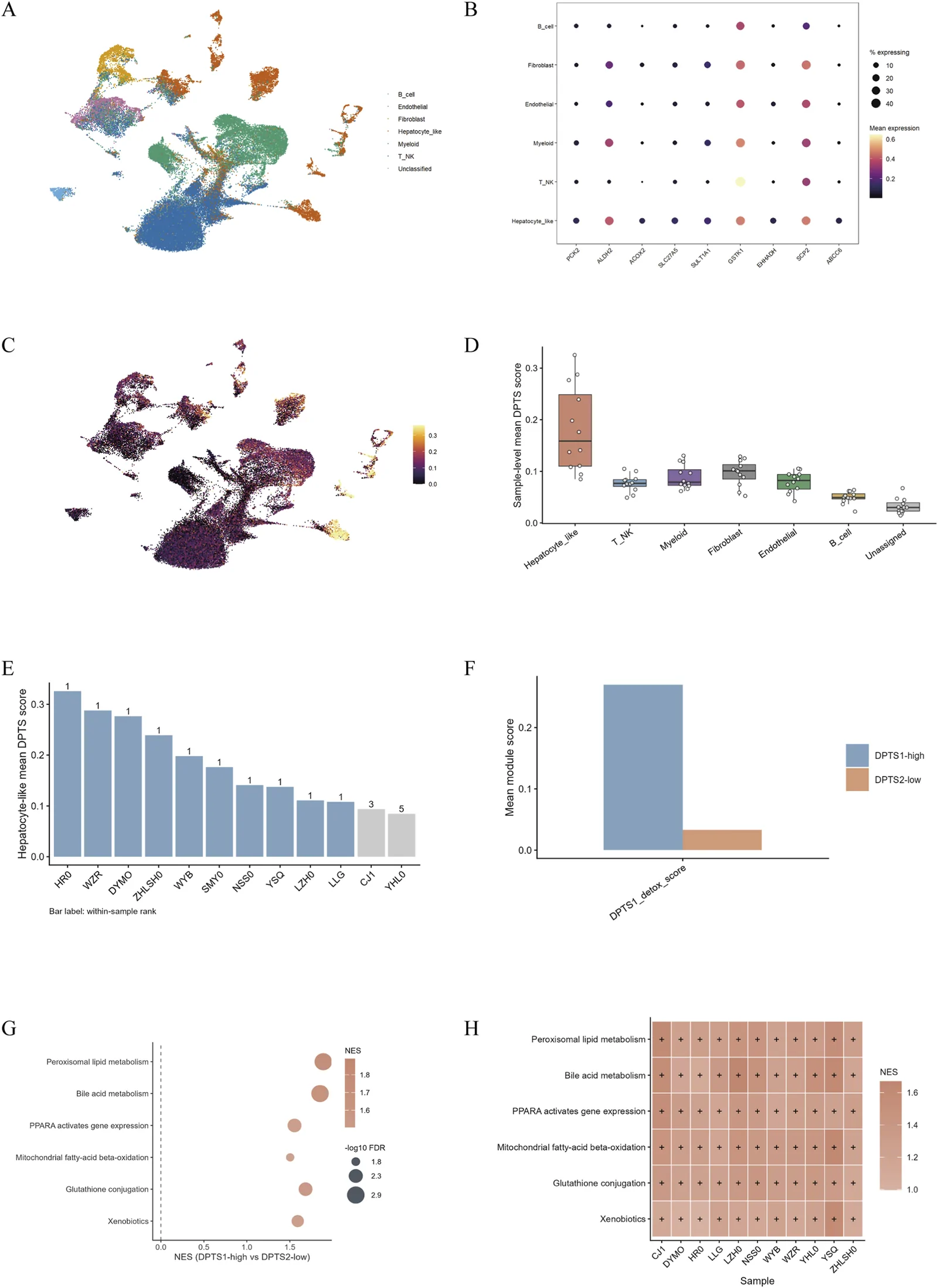
Sample-aware single-cell localization and pathway characterization of the DPTS program. **(A)** UMAP of GSE290925 cells coloured by marker-based inferred cell type. **(B)** Expression of selected DPTS candidate genes across inferred cell types. Dot size represents the percentage of expressing cells, and colour represents mean log-normalized expression. **(C)** Distribution of the DPTS detoxification score across the UMAP. **(D)** Sample-level mean DPTS scores across inferred cell types. Each point represents one biological sample. **(E)** Mean hepatocyte-like DPTS score and its within-sample rank across the 12 samples. **(F)** DPTS detoxification scores in hepatocyte-like cells divided descriptively into median-defined DPTS-high and DPTS-low states. **(G)** Reactome pathway enrichment in sample-adjusted pseudobulk comparisons of DPTS-high versus DPTS-low hepatocyte-like cells. **(H)** Sample-specific normalized enrichment scores for the six prespecified pathways. Positive values indicate enrichment in the DPTS-high state.

### 3.5 Spatial coordination of DPTS-associated expression, candidate genes, and metabolic programs

The CHC20 and CHC23 specimens contained 2,261 and 1,987 in-tissue spots, respectively, with acceptable quality distributions and marked spatial heterogeneity (Figure 6A; Supplementary Figure S5A). The no-core DPTS and core-target scores formed partially overlapping spatial patterns in both specimens (Figures 6B,C). All nine candidate genes were detected in both specimens, with detection rates ranging from 62.9% to 99.9% of spots; spatial maps of ALDH2, EHHADH, and PCK2 and the extended target analysis confirmed heterogeneous expression across tissue regions (Figure 6D; Supplementary Figures S5B–F). The no-core DPTS score was positively correlated with the core-target score in CHC20 (rho = 0.516) and CHC23 (rho = 0.308; Figure 6E). Adjustment for hepatocyte-like identity and technical covariates attenuated these associations, but modest positive correlations remained in CHC20 (partial rho = 0.161, P = 1.32 × 10^^−14^) and CHC23 (partial rho = 0.186, P = 6.61 × 10^^−17^; Figure 6F; Supplementary Figure S5G). The adjusted no-core DPTS score was positively associated with the hepatocyte-like score and inversely associated with immune, stromal, and MIF-CD74-CD44 scores in both specimens, whereas its association with the FN1-integrin-CD44 score was weaker and less consistent (Supplementary Figure S5G). All five candidate-derived metabolic proxy scores showed concordant positive spatial correlations with the no-core DPTS score across the two specimens (rho = 0.148–0.369; Figures 6G,H). These findings indicated exploratory within-section spatial coordination between DPTS-associated metabolic expression and the prioritized genes. They did not constitute subtype-level spatial validation because only two specimens were available and neither specimen had an independently assigned DPTS status.

**FIGURE 6:**
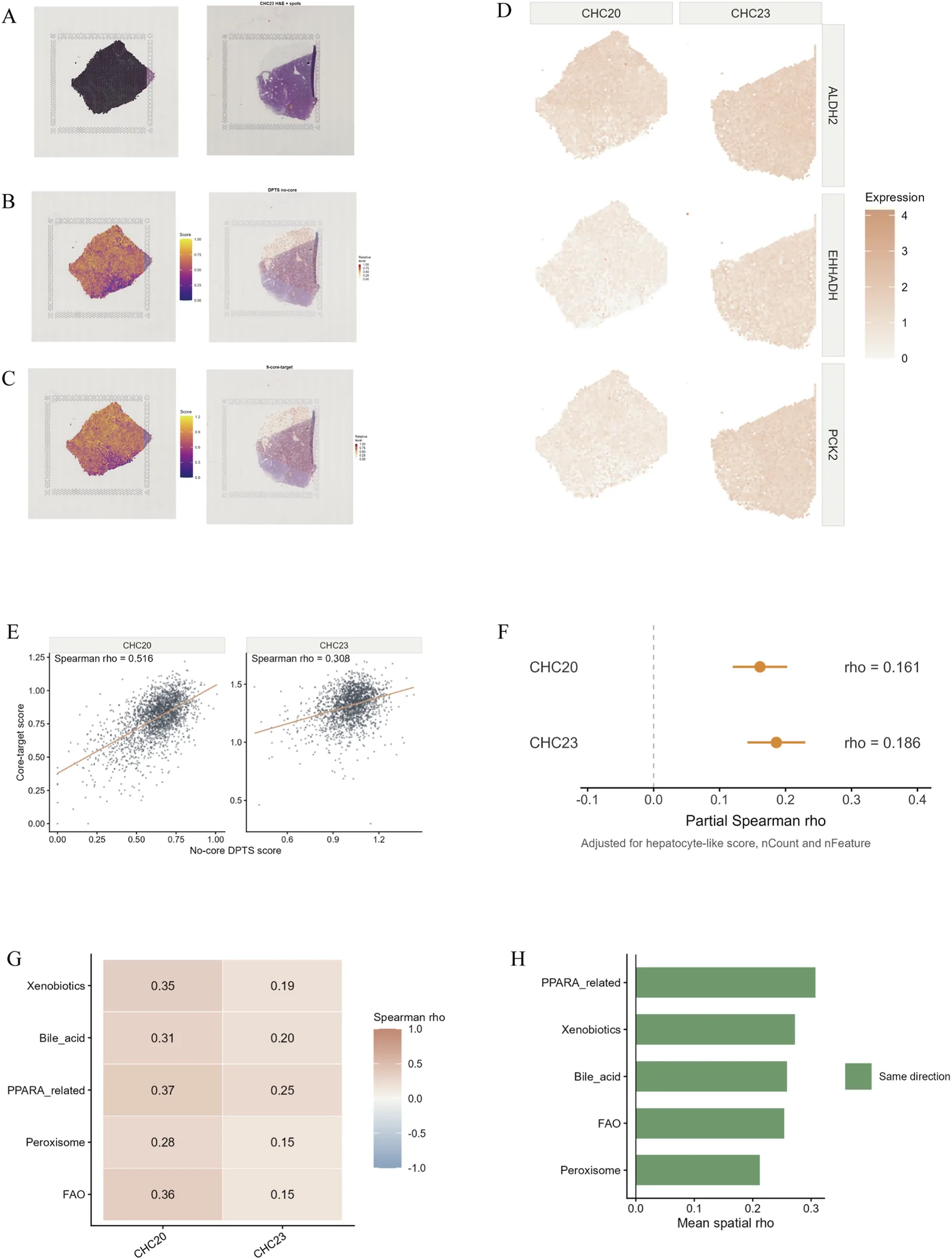
Spatial colocalization of DPTS-associated metabolic programs and candidate targets in HCC. **(A)** Histological images and Visium spot distributions for CHC20 and CHC23. Reconstructed from publicly available spatial transcriptomic data deposited in GEO under accession GSE245908 (samples GSM7850822 and GSM7850823), associated with Giraud et al., *Cell Reports* 43, 113773 (2024), doi:10.1016/j.celrep.2024.113773. The image was reconstructed by the authors using the deposited tissue images and Visium spot-coordinate files. Source: https://www.ncbi.nlm.nih.gov/geo/query/acc.cgi?acc=GSE245908. **(B)** Spatial distribution of the no-core DPTS score, calculated after excluding the nine prioritized targets. **(C)** Spatial distribution of the nine-gene core-target score. **(D)** Spatial expression of ALDH2, EHHADH, and PCK2. **(E)** Raw spot-level Spearman correlations between the no-core DPTS and core-target scores. **(F)** Partial Spearman correlations after adjustment for the hepatocyte-like score, nCount_Spatial, and nFeature_Spatial. **(G)** Spearman correlations between the no-core DPTS score and candidate-derived fatty-acid oxidation, peroxisomal, PPARA-related, bile-acid, and xenobiotic metabolic proxy scores. **(H)** Mean correlation coefficients and cross-specimen directional concordance for the five metabolic proxy scores. Correlation analyses were performed separately for each specimen. Spot-level P values are exploratory because of spatial autocorrelation and the availability of only two specimens. Neither specimen had an independently assigned DPTS status; therefore, these analyses represent within-section spatial associations rather than subtype-level spatial validation.

### 3.6 HCC-restricted pathway analyses support detoxification and metabolic heterogeneity across DPTS-like tumors

After restricting the external validation cohorts to HCC samples, 341 TCGA-LIHC tumors and 163 ICGC-LIRI-JP tumors were retained for pathway-level analysis. Reactome preranked GSEA showed that peroxisomal lipid metabolism, bile acid metabolism, mitochondrial fatty-acid beta-oxidation, glutathione conjugation and xenobiotic-related programs were preferentially aligned with the DPTS1-like phenotype in both cohorts, whereas PPARA-related enrichment was weaker and less consistent across cohorts (Figure 7B). Consistent with these enrichment patterns, representative custom module scores for xenobiotic phase-I metabolism, phase-II conjugation, bile acid metabolism and fatty-acid oxidation differed between DPTS1-like and DPTS2-like tumors across the two bulk cohorts (Figure 7C). Comparison with hepatocyte-like pseudobulk Reactome enrichment further supported cross-modal concordance for selected detoxification and lipid-metabolic programs, while indicating that these findings reflect pathway-level transcriptomic agreement rather than direct exposure measurement (Figure 7D). Together with the workflow, spatial/single-cell evidence, candidate pathway network and evidence matrix shown in Figures 7A,E–G, these results support a reproducible detoxification- and metabolism-related transcriptomic axis associated with DPTS-like heterogeneity in HCC. Supplementary analyses further characterized the robustness and boundaries of these pathway-level associations. Most predefined custom modules showed similar directions across TCGA-LIHC and ICGC-LIRI-JP. Angiogenesis showed a weak, non-significant positive difference in TCGA-LIHC but a larger positive difference in ICGC-LIRI-JP (Supplementary Figure S6A). Custom preranked GSEA and module-score comparisons provided complementary evidence for cohort-level pathway shifts but also showed that not all immune or stromal programs were uniformly reproduced across datasets (Supplementary Figures S6B,C). Exploratory correlation analyses linked metabolic, immune and stromal pathway scores with predefined bulk ligand-receptor axis scores, identifying sign-consistent pathway-axis associations across cohorts (Supplementary Figures S6D,E). However, because these axes were inferred from bulk expression signatures rather than spatially resolved ligand-receptor measurements, they should be interpreted as bulk transcriptomic associations related to crosstalk programs, not as proof of active cell-cell communication (Supplementary Figure S6F).

**FIGURE 7:**
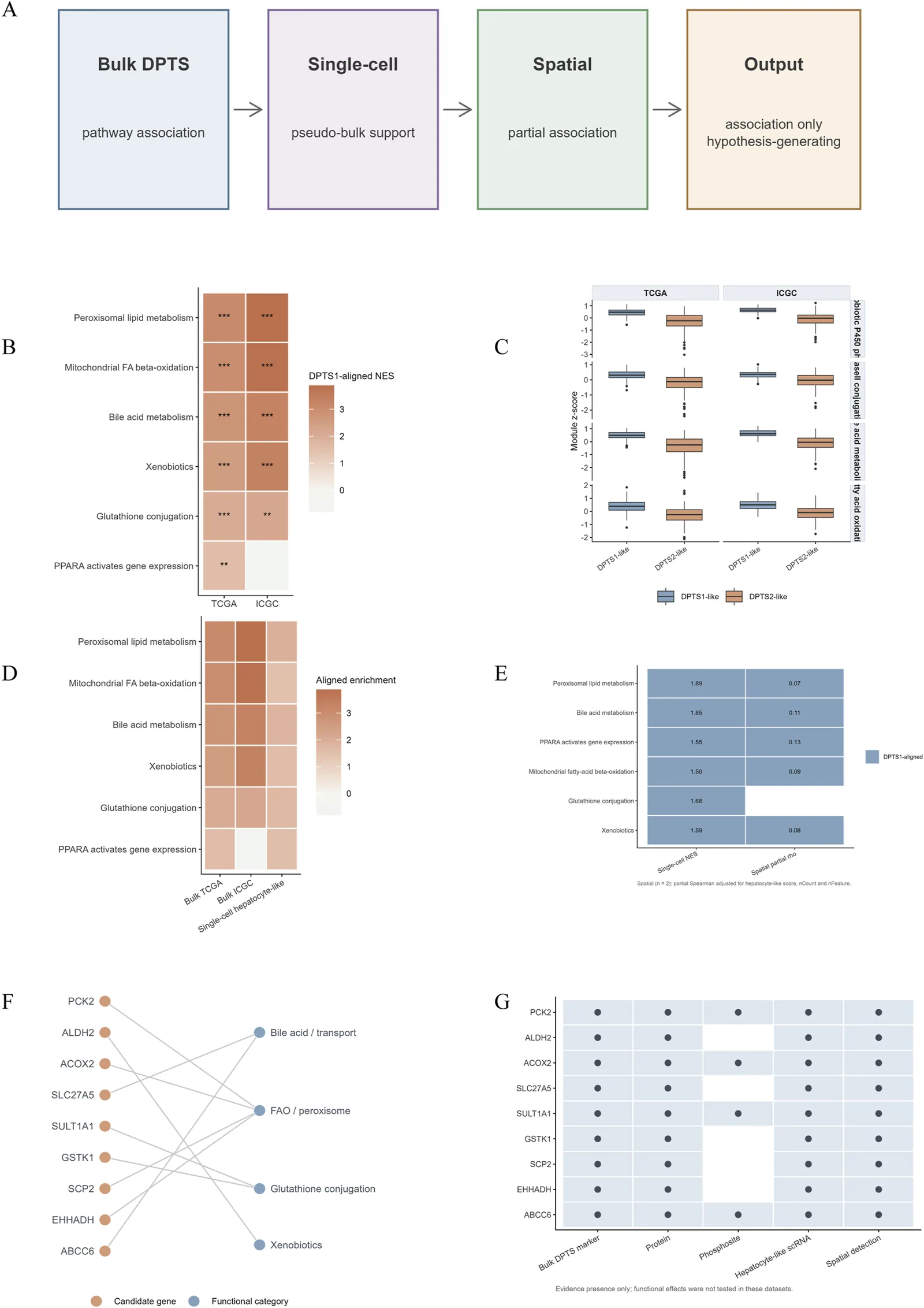
Cross-cohort and cross-modal characterization of detoxification- and metabolism-related pathway heterogeneity in DPTS-like HCC. **(A)** Overview of the cross-modal analysis framework used to evaluate DPTS-associated pathway programs across bulk transcriptomic, single-cell/pseudobulk and spatial evidence layers. **(B)** Heatmap showing selected Reactome preranked GSEA results in HCC-restricted TCGA-LIHC and ICGC-LIRI-JP cohorts. Values are displayed as DPTS1-aligned normalized enrichment scores (NES); asterisks denote adjusted P values: *FDR < 0.05, **FDR < 0.01, and ***FDR < 0.001. **(C)** Representative custom pathway/module scores for xenobiotic phase-I metabolism, phase-II conjugation, bile acid metabolism and fatty-acid oxidation in DPTS1-like and DPTS2-like tumors across the two HCC cohorts. **(D)** Comparison of selected Reactome enrichment patterns between bulk HCC cohorts and hepatocyte-like pseudobulk single-cell evidence. **(E)** Spatial and single-cell cross-evidence summary for DPTS-associated pathway programs. **(F)** Candidate pathway network summarizing selected detoxification- and metabolism-related modules supported by the analyses. **(G)** Evidence matrix summarizing the level of support across transcriptomic and cross-modal evidence layers. The external cohort analyses were restricted to HCC samples and should be interpreted as transcriptomic pathway evidence rather than direct measurement of DEHP exposure, ligand-receptor activity, or protein/phosphoprotein validation.

### 3.7 Computational candidate prioritization and experimental characterization of DEHP-associated cellular responses

LINCS-based reversal screening ranked PD-0325901, Trametinib, and Regorafenib as the three highest-priority compounds for follow-up, based on their HepG2 perturbation signatures and overlap with DPTS2-associated metabolic and detoxification genes (Supplementary Figures S7A,B). The nine docking pairs produced predicted affinities ranging from −6.34 to −10.89 kcal/mol (Supplementary Figure S7C). Regorafenib showed the most negative predicted affinity for EHHADH (−10.89 kcal/mol) and also showed negative predicted affinities for PCK2 (−10.56 kcal/mol) and ALDH2 (−10.35 kcal/mol). Trametinib showed a target-dependent pattern, with a more negative predicted affinity for ALDH2 (−10.35 kcal/mol) than for EHHADH (−6.34 kcal/mol), whereas PD-0325901 showed values from −7.45 to −8.02 kcal/mol. These results were used only to prioritize computational candidates and did not demonstrate direct binding or drug efficacy. In the cell experiments, the CCK-8 assay established the time- and concentration-dependent response profile of LO2, Huh7, and Hep-3B cells and supported the use of 25 μM DEHP for downstream assays (Figure 8A). Compared with LO2 cells, Huh7 cells showed lower basal mRNA levels of ALDH2, EHHADH, and PCK2, with Holm-adjusted P values of 0.0115, 0.00665, and 0.00233, respectively, whereas Hep-3B cells showed lower ALDH2 and EHHADH expression, with adjusted P values of 0.0115 and 0.0103; the PCK2 comparison in Hep-3B cells was not significant (adjusted P = 0.0859) (Figure 8B). The immunoblot analyses included ALDH2 and EHHADH only, with representative immunoblots shown under vehicle, 10 μM, and 25 μM conditions (Figure 8C; Supplementary Figures S8A,B). Exposure to 25 μM DEHP increased the crystal violet-positive area in Transwell assays and increased the EdU-positive area ratio in both Huh7 and Hep-3B cells (Figures 8D,E; Supplementary Figure S8C). Thus, the current experiments support DEHP-associated changes in cellular migration and DNA-synthesis readouts and expression of selected detoxification-related genes, but they do not directly validate the predicted binding of PD-0325901, Trametinib, or Regorafenib to ALDH2, PCK2, or EHHADH.

**FIGURE 8:**
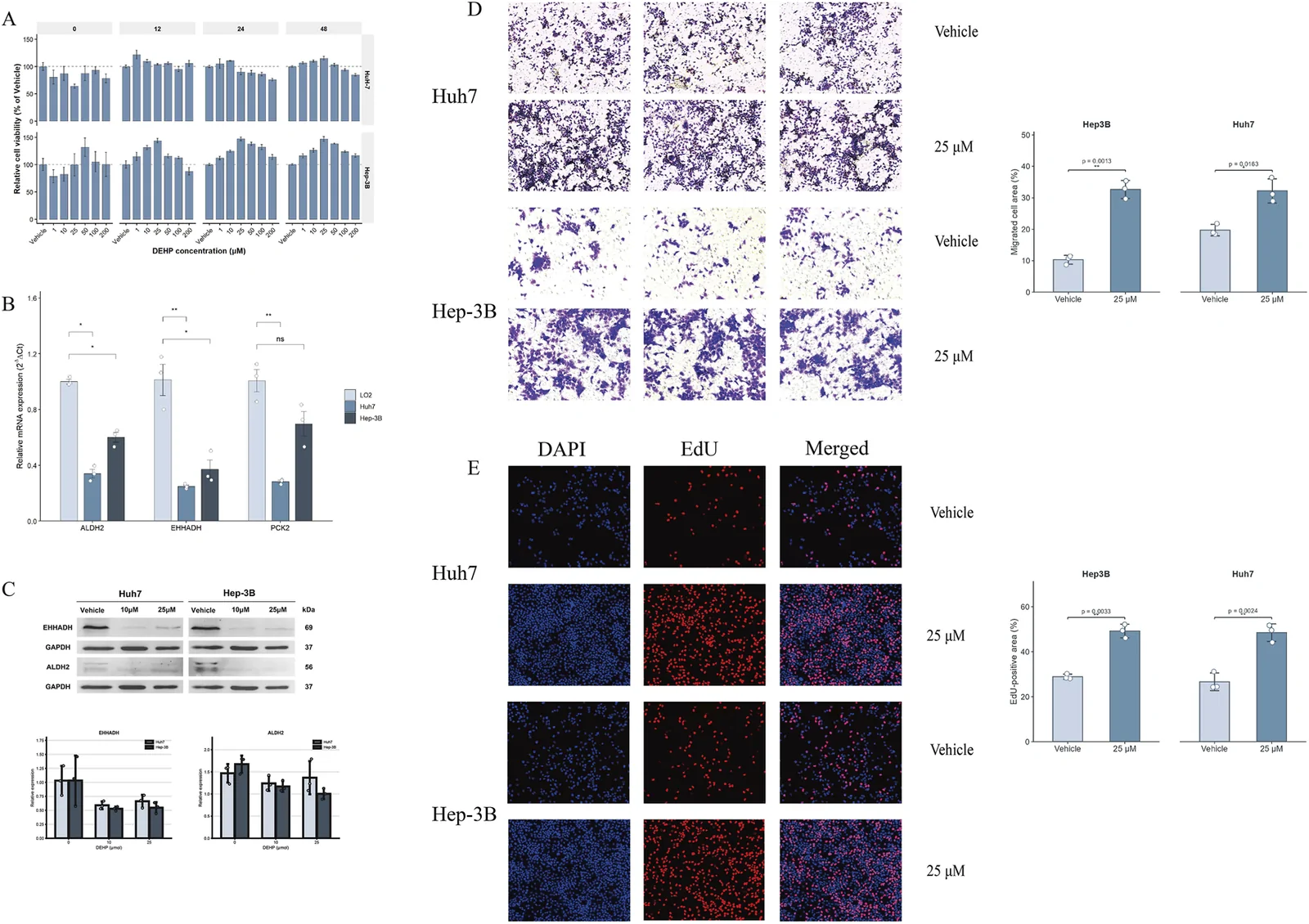
Experimental characterization of DEHP-associated cellular responses. **(A)** CCK-8 measurements in LO2, Huh7, and Hep-3B cells at 0, 12, 24, and 48 h under vehicle and 25, 50, 100, and 200 μM DEHP conditions. Values are shown relative to the vehicle group. **(B)** Basal relative mRNA expression of ALDH2, EHHADH, and PCK2 in LO2, Huh7, and Hep-3B cells. Expression was calculated using the 2^^−ΔΔCt^ method; statistical comparisons were performed on ΔCt values with Holm correction. **(C)** Representative immunoblots and densitometric quantification of ALDH2 and EHHADH in Huh7 and Hep-3B cells treated with vehicle, 10 μM DEHP, or 25 μM DEHP, with GAPDH as the loading control. ALDH2 and EHHADH were detected on the same membrane using the same sample set; therefore, the same GAPDH loading-control image is shown for both target panels. This reuse of the loading-control image is disclosed for transparency and does not represent additional biological replicates. Densitometric quantification was performed using independent biological replicates. PCK2 was not assessed by Western blotting. **(D)** Representative Transwell images and image-based quantification of migrated Huh7 and Hep-3B cells under vehicle and 25 μM DEHP conditions. **(E)** Representative DAPI, EdU, and merged images and corresponding quantification of EdU incorporation in Huh7 and Hep-3B cells under vehicle and 25 μM DEHP conditions. Image-based quantifications were obtained from three fields per condition. The experimental results describe cellular responses to DEHP exposure *in vitro* and do not establish effects of the computationally prioritized compounds.

## 4 Discussion

This study supports a conceptual shift from an exposure-to-cancer causal model toward the characterization of DEHP-relevant detoxification and metabolic heterogeneity in HCC. Our Mendelian randomization analysis did not provide robust evidence that genetically proxied MECPP or MEHHP was causally associated with HCC. MR can reduce confounding and reverse causation, but its interpretation depends on instrument strength, variant independence, exclusion-restriction assumptions, and the availability of sufficiently informative genetic instruments (Larsson et al., 2023; Zuber et al., 2022). In the present analysis, the limited number and strength of the available instruments, particularly for MEHHP, restricted causal inference. Therefore, the negative HCC findings should neither be interpreted as evidence that DEHP is biologically irrelevant to the liver nor be used to support a DEHP-HCC causal relationship. Instead, they motivate the examination of downstream detoxification and metabolic programs as associative features of HCC heterogeneity.

The toxicological evidence for DEHP is characterized by an important asymmetry between experimental-animal and human data. Dietary DEHP exposure has produced hepatocellular adenomas and carcinomas in rats and mice, and recent NTP studies reported clear evidence of carcinogenic activity in both male and female rats (National Toxicology Program, 2021b; National Toxicology Program, 1982). Accordingly, DEHP is listed as reasonably anticipated to be a human carcinogen on the basis of sufficient evidence from experimental animals (National Toxicology Program, 2021a). However, the same authoritative assessment concluded that epidemiological data were inadequate to evaluate cancer risk specifically associated with human DEHP exposure (National Toxicology Program, 2021a). Recent human studies instead provide mainly cross-sectional evidence linking DEHP metabolites or broader phthalate exposure to NAFLD or MASLD, without establishing temporality or causality (He et al., 2023; Gogola et al., 2025). Moreover, differences between rodents and humans in DEHP metabolism, PPARA-related responses, hepatocyte proliferation, and non-PPARA pathways limit direct extrapolation of rodent liver tumor findings to human HCC (Rusyn et al., 2006; Rusyn and Corton, 2012; Ito et al., 2014). Thus, the available evidence supports the biological plausibility of DEHP-related hepatic metabolic disruption, but it does not demonstrate that DEHP causes human HCC or that the DPTS states identified here were generated by DEHP exposure.

The DPTS classification is therefore best understood as a detoxification- and metabolism-related classification of HCC rather than as an exposure-defined subtype. DPTS2 was characterized by lower xenobiotic metabolism, bile acid handling, fatty-acid oxidation, phase II conjugation, and transporter-related activity. This pattern is compatible with the extensive metabolic reprogramming and loss of differentiated hepatic functions observed during HCC progression (Park and Hall, 2025). Previous proteogenomic studies have similarly identified HCC subtypes distinguished by metabolic reprogramming, microenvironmental features, kinase activity, prognosis, and treatment response (Xing et al., 2023; Li B. et al., 2024). The HCC-restricted TCGA-LIHC and ICGC-LIRI-JP analyses further supported cross-cohort transcriptomic alignment of selected detoxification, bile acid, peroxisomal lipid, mitochondrial fatty-acid oxidation, glutathione-conjugation, and xenobiotic-related programs. However, the weaker cross-cohort behavior of PPARA-related enrichment and the discordant angiogenesis module indicate that the DPTS-associated pathway pattern is not uniform across all biological programs. An equally plausible interpretation is that DPTS2 reflects a general hepatocyte metabolic-identity-low state rather than a DEHP-specific toxicological imprint. The association between DPTS2-like status and poorer survival in TCGA-LIHC, together with its non-replication in ICGC-LIRI-JP, further indicates that its prognostic relevance remains cohort-dependent and requires independent validation. Importantly, the TCGA-LIHC and ICGC-LIRI-JP analyses were based on transcriptomic projection of a CPTAC-derived proteomic and phosphoproteomic classification. Cross-platform projection may introduce measurement and signature-transfer bias and should not be interpreted as independent proteomic or phosphoproteomic replication. Validation in an external cohort with matched proteomic and phosphoproteomic measurements remains necessary.

The prioritized genes provide biological anchors for the DPTS phenotype, but they should not be regarded as established drivers or DEHP-specific targets. ALDH2 participates in mitochondrial aldehyde detoxification and redox regulation, and experimental work has linked ALDH2 expression to ROS/Nrf2-related autophagy and immune escape in HCC models (Zhang and Fu, 2021; Hu et al., 2022). PCK2 has been associated with HCC metabolism, prognosis, immune-related features, proliferation, and migration, although these observations do not establish its role in DEHP responses (Li C. et al., 2024). EHHADH is a peroxisomal fatty-acid metabolism enzyme, and the miR-1290-EHHADH axis has been linked to HCC cell proliferation (Lee et al., 2024). SLC27A5 has been reported to regulate fatty-acid-related functions, ferroptosis, sorafenib response, and metastatic behavior in HCC (Xu et al., 2023; Nie et al., 2024). ABCC6 knockdown has also been associated with increased HCC cell proliferation through PPARA-related regulation (Zhao et al., 2022). These studies support the biological relevance of the candidate set, but our heuristic prioritization, expression analyses, and evidence-presence summaries do not establish causal regulation.

The single-cell and spatial analyses indicate that DPTS-associated genes and pathways are preferentially detected in hepatocyte-like compartments. Single-cell studies have demonstrated extensive heterogeneity among malignant hepatocyte-like, immune, stromal, and endothelial populations in primary and metastatic HCC (Lu et al., 2022). Spatial studies have further shown that immune-suppressive populations and treatment-associated programs occupy distinct tissue regions (Qiu et al., 2024; Zhang et al., 2023). In our data, the enrichment of the DPTS program in hepatocyte-like cells and its modest spatial coordination with the candidate-target score support a hepatic metabolic origin for the bulk signal. Nevertheless, cell identities were inferred from marker modules rather than reference-mapped annotations. Moreover, although CHC20 and CHC23 were confirmed as HCC tumor sections, sample-specific clinical characteristics and independent DPTS assignments were unavailable. The two-specimen sample size and spatial dependence among neighboring spots further limited generalization. Accordingly, these results support exploratory spatial localization of related metabolic expression programs but cannot establish malignant-cell specificity, subtype-level spatial validation, individual DEHP exposure, or causal spatial interactions.

The immune and stromal findings should also be interpreted as associative rather than mechanistic. The HCC immune microenvironment is an important determinant of tumor progression and response to immune-based therapy (Donne and Lujambio, 2023; Shen et al., 2024). Spatially organized immune barriers and immunosuppressive cellular states have been associated with heterogeneous immunotherapy responses (Qiu et al., 2024; Liu et al., 2023). In our study, DPTS-associated metabolic scores were related to bulk immune/stromal expression axes and showed spatial associations with selected immune and stromal scores. The Supplementary Figure S6 analyses further linked metabolic, immune, and stromal pathway scores with predefined bulk ligand-receptor axis scores across the HCC-restricted validation cohorts. However, these measurements represent pathway-score correlation, bulk axis-score association, and spatial co-localization patterns rather than direct ligand-receptor activity or experimentally demonstrated cell-cell communication. They therefore cannot establish whether detoxification suppression drives microenvironmental remodeling, results from tumor dedifferentiation within immune-rich regions, or simply accompanies both processes.

The computational drug-prioritization results require an equally restricted interpretation. Regorafenib has an established role in the systemic treatment sequence for selected patients with advanced HCC, but this clinical activity does not imply that ALDH2, PCK2, or EHHADH are its relevant targets in the present setting (Gordan et al., 2024). Molecular docking is useful for structure-based candidate prioritization, but predicted scores and binding poses are sensitive to receptor preparation, binding-site definition, protein flexibility, protonation states, and scoring-function limitations (Stanzione et al., 2021). None of these compounds was tested in the cell experiments. Likewise, the basal qPCR results and the DEHP-associated increases in Transwell and EdU image-based readouts support expression and phenotypic associations but do not demonstrate target engagement, therapeutic efficacy, or mediation of the DEHP response by ALDH2, EHHADH, or PCK2.

Several limitations define the scope of these conclusions. First, the MR analysis was constrained by weak or sparse instruments, and the independence of the selected variants could not be independently reconstructed from the available records. Second, the CPTAC, TCGA-LIHC, ICGC-LIRI-JP, single-cell, and spatial transcriptomic datasets did not contain individual-level DEHP exposure measurements. Consequently, neither the DPTS classification nor its bulk, single-cell, and spatial distributions can be attributed to prior DEHP exposure; DPTS should be interpreted as a DEHP-relevant detoxification and metabolic phenotype rather than an exposure biomarker, a DEHP-induced subtype, or a DEHP-specific carcinogenic mechanism. Third, the HCC-restricted external pathway analyses provide transcriptomic support only. They do not provide independent protein- or phosphosite-level validation of DPTS, and the bulk ligand-receptor axis analyses do not constitute direct evidence of ligand-receptor activity or cell-cell communication. Fourth, the prognostic association was observed in TCGA-LIHC but was not replicated in ICGC-LIRI-JP, where the number of events and the size of the DPTS1-like group were limited. Fifth, the marker-based cell identities, absence of sample-specific clinical information and independent DPTS assignments, two-specimen spatial sample size, and spatial dependence among neighboring spots limited generalization and precluded subtype-level spatial validation. Sixth, the cell experiments were phenotypic and did not include genetic perturbation, rescue experiments, metabolomic measurements, biochemical binding assays, or pharmacological testing of the docking compounds. Finally, Western blotting was limited to ALDH2 and EHHADH and should not be interpreted as protein-level validation of PCK2 or of the full candidate set.

In conclusion, our findings define a detoxification-suppressed and metabolically altered HCC molecular phenotype that is relevant to biological pathways affected by DEHP but is not demonstrated to be caused by DEHP exposure. CPTAC proteomic and phosphoproteomic data defined the DPTS classification, whereas the TCGA-LIHC and ICGC-LIRI-JP analyses provided HCC-restricted transcriptomic projection and pathway-level support rather than independent protein- or phosphosite-level replication. The single-cell and spatial analyses supported exploratory localization of related detoxification and metabolic expression programs, and the bulk axis-score analyses provided additional associative links to immune and stromal expression programs without proving direct cell-cell communication. The survival, candidate-gene, docking, and cellular findings remained hypothesis-generating. This bounded interpretation reconciles the negative MR results with the downstream multi-omics and experimental analyses and provides a testable framework for future exposure-informed cohorts, matched proteomic and phosphoproteomic validation, spatial ligand-receptor studies, genetic perturbation, metabolomic profiling, target-engagement experiments, and pharmacological validation.

## Data Availability

Publicly available datasets were analyzed in this study. This data can be found here: GWAS Catalog: MECPP (GCST90319479), https://www.ebi.ac.uk/gwas/studies/GCST90319479; MEHHP (GCST90319480), https://www.ebi.ac.uk/gwas/studies/GCST90319480. FinnGen release 12: NAFLD (NAFLD), https://storage.googleapis.com/finngen-public-data-r12/summary_stats/release/finngen_R12_NAFLD.gz; liver fibrosis and cirrhosis (K11_FIBROCHIRLIV), https://storage.googleapis.com/finngen-public-data-r12/summary_stats/release/finngen_R12_K11_FIBROCHIRLIV.gz; toxic liver disease (K11_TOXLIV), https://storage.googleapis.com/finngen-public-data-r12/summary_stats/release/finngen_R12_K11_TOXLIV.gz; hepatocellular carcinoma (C3_HEPATOCELLU_CARC_EXALLC), https://storage.googleapis.com/finngen-public-data-r12/summary_stats/release/finngen_R12_C3_HEPATOCELLU_CARC_EXALLC.gz. UK Biobank Neale Lab Round 2: ALT (30620), AST (30650), ALP (30610), GGT (30730), and total bilirubin (30840), http://www.nealelab.is/uk-biobank. Proteomic Data Commons: PDC000198, https://pdc.cancer.gov/pdc/study/PDC000198; PDC000199, https://pdc.cancer.gov/pdc/study/PDC000199. HCCDB: TCGA-LIHC (HCCDB15) and ICGC-LIRI-JP (HCCDB18), http://lifeome.net/database/hccdb/home.html. Gene Expression Omnibus: GSE290925, https://www.ncbi.nlm.nih.gov/geo/query/acc.cgi?acc=GSE290925; GSE245908, https://www.ncbi.nlm.nih.gov/geo/query/acc.cgi?acc=GSE245908. Protein structures: RCSB PDB 1O02, https://www.rcsb.org/structure/1O02; AlphaFold Q16822, https://alphafold.ebi.ac.uk/entry/Q16822; AlphaFold Q08426, https://alphafold.ebi.ac.uk/entry/Q08426. PubChem: https://pubchem.ncbi.nlm.nih.gov/. CMap/LINCS: https://clue.io/.

## Supplementary material

SUPPLEMENTARY FIGURE S1 Instrument-level and sensitivity analyses for the MR results. **(A,B)** SNP-level estimates for FinnGen and UK Biobank outcomes. **(C)** Instrument-strength assessment. **(D)** Comparison of inverse-variance-weighted, weighted-median, and MR-Egger estimates. **(E)** Heterogeneity and MR-Egger intercept analyses. **(F,G)** Leave-one-out analyses for FinnGen and UK Biobank outcomes.

SUPPLEMENTARY FIGURE S2 Input composition and robustness analyses for DPTS classification. **(A)** Numbers of protein and phosphosite features across the functional categories used for subtype discovery. **(B)** Effect directions and false discovery rates for the complete set of subtype-associated features. **(C)** Distribution of DPTS1-associated and DPTS2-associated marker features across functional categories. **(D)** Proportion of ambiguous clustering, mean silhouette width, and combined rank for K = 2 to K = 6. **(E)** Consensus matrices for K = 2 to K = 6 displayed using a common sample order. **(F)** Sample-level consensus with the original subtype and the alternative subtype across 300 feature-resampling analyses. **(G)** Distributions of pairwise consensus values within and between the original DPTS subtypes. Each feature-resampling analysis used 80% of the input features and Ward.D2 hierarchical clustering with K = 2.

SUPPLEMENTARY FIGURE S3 Molecular evidence, candidate ranking, and sensitivity analyses for external DPTS projection. **(A,B)** Complete protein-level and phosphosite-level differences between DPTS2 and DPTS1. **(C)** Full multi-evidence heuristic priority ranking, with the ten leading scores labelled. **(D)** Cox regression sensitivity analyses using projected DPTS groups and the continuous DPTS suppression score in TCGA-LIHC and ICGC-LIRI-JP. **(E)** Proportional-hazards diagnostics and the adjusted TCGA log-time interaction analysis. **(F)** Numbers of DPTS1-like and DPTS2-like tumors and survival events in the external cohorts. **(G)** Overlap among the molecular evidence sources used for candidate prioritization. **(H)** Functional classification of the candidate-gene set. ICGC results are external estimates and are not interpreted as replicated effects.

SUPPLEMENTARY FIGURE S4 Sample-level quality control and robustness summaries for the single-cell analysis. **(A)** Numbers of cells, median RNA counts, median detected genes, and median mitochondrial transcript percentages for each biological sample. **(B)** Inferred cell-type composition of the 12 samples. **(C)** Expression of the complete DPTS candidate-gene set across inferred cell types. **(D)** Distribution of sample-level mean DPTS scores across all inferred cell types. Each point represents one sample and cell-type combination. **(E)** Heatmap of mean DPTS scores for each sample and inferred cell type. **(F)** Sample-level hepatocyte-like DPTS scores and their within-sample ranks. Cell-type assignments were inferred from marker-module scores and should not be interpreted as independently validated annotations.

SUPPLEMENTARY FIGURE S5 Quality assessment and extended spatial analysis of DPTS-associated targets. **(A)** Distributions of nCount_Spatial and nFeature_Spatial in CHC20 and CHC23. **(B)** Spatial expression of PCK2, ALDH2, and ACOX2 in CHC20. **(C)** Spatial expression of SLC27A5, SULT1A1, and GSTK1 in CHC20. **(D)** Spatial expression of SCP2, EHHADH, and ABCC6 in CHC20. **(E)** Representative core-target expression maps in CHC23. **(F)** Percentage of spots with detectable expression of each of the nine candidate genes in both specimens. **(G)** Raw and partial Spearman correlations of the no-core DPTS score with the core-target, hepatocyte-like, immune, stromal, MIF-CD74-CD44, and FN1-integrin-CD44 scores. Partial correlations were adjusted for nCount_Spatial and nFeature_Spatial and, except for the hepatocyte-like analysis, the hepatocyte-like score. Analyses are exploratory and represent two spatial specimens.

SUPPLEMENTARY FIGURE S6 Supplementary HCC-restricted pathway and exploratory bulk axis-score analyses. **(A)** Cross-cohort consistency of predefined custom pathway/module score differences between DPTS1-like and DPTS2-like tumors in TCGA-LIHC and ICGC-LIRI-JP. Angiogenesis showed a weak, non-significant positive difference in TCGA-LIHC but a larger positive difference in ICGC-LIRI-JP. **(B)** Custom preranked GSEA normalized enrichment scores across the two HCC cohorts. **(C)** Module-score differences for all predefined custom pathways in the HCC-restricted validation cohorts. **(D)** Exploratory correlations between metabolic pathway scores and predefined bulk ligand-receptor axis scores. **(E)** Exploratory correlations between immune/stromal pathway scores and predefined bulk ligand-receptor axis scores. **(F)** Differences in predefined bulk ligand-receptor axis scores between DPTS1-like and DPTS2-like tumors across cohorts. These analyses summarize bulk transcriptomic axis-score associations and do not constitute direct evidence of ligand-receptor activity or cell-cell communication.

SUPPLEMENTARY FIGURE S7 Computational drug-reversal prioritization and molecular docking. **(A)** Priority ranking of the top ten compounds identified by the LINCS-based reversal analysis. **(B)** Evidence matrix summarizing reversal-direction evidence, HepG2 evidence, mechanistic annotation, and docking priority. **(C)** Predicted docking affinities for PD-0325901, Trametinib, and Regorafenib against ALDH2, PCK2, and EHHADH. Values are reported in kcal/mol; more negative values indicate more favorable predicted scores within the docking model. **(D)** Representative predicted binding poses for the nine compound-target pairs. These structural poses are computational predictions and do not demonstrate experimentally confirmed binding or target engagement.

SUPPLEMENTARY FIGURE S8 Representative immunoblot and EdU images. **(A)** Representative EHHADH immunoblots in Huh7 and Hep-3B cells treated with vehicle, 10 μM DEHP, or 25 μM DEHP, with GAPDH as the loading control. **(B)** Representative ALDH2 immunoblots under the same conditions, with GAPDH as the loading control. ALDH2 and EHHADH were detected on the same membrane using the same sample set; accordingly, the same GAPDH loading-control image is shown in panels A and B. This reuse of the loading-control image is for illustrative purposes and does not represent additional biological replicates. Densitometric quantification was performed using independent biological replicates. **(C)** Representative DAPI, EdU, and merged images from Huh7 and Hep-3B cells treated with vehicle or 25 μM DEHP. These images provide experimental characterization of selected protein and EdU-associated readouts; they do not constitute validation of the molecular-docking predictions.
