# Supplementary material for: Genetic evidence and multi-omics characterization of DEHP-relevant detoxification and metabolic heterogeneity in hepatocellular carcinoma
Source: Front Toxicol. 2026 Sep 29;8:1925431. doi: 10.3389/ftox.2026.1925431 (PMC13659102; doi:10.3389/ftox.2026.1925431)
Supplement: Supplementary file 1 [file Supplementaryfile1.zip › Supplementary Figure/Supplementary Table SF1.docx]

**Supplementary Table 1. Instrument strength and sensitivity analyses for Mendelian randomization**

**A. Instrument strength summary**

| **Exposure** | **SNP instruments** | **n** | **F statistics** | **Min / median / mean F** | **Weak instruments** | **Methodological interpretation** |
| --- | --- | --- | --- | --- | --- | --- |
| MECPP | rs1359232, rs117529685, rs117033379, rs4918798 | 4 | 9, 121, 144, 81 | 9 / 101 / 88.75 | 1/4 (rs1359232, F = 9) | Exploratory four-SNP MR; one instrument had F = 9. MR-Egger estimates were imprecise because only two residual degrees of freedom were available, and LD independence could not be verified because clumping metadata were unavailable. |
| MEHHP | rs7895726 | 1 | 9 | 9 / 9 / 9.00 | 1/1 (rs7895726, F = 9) | Single-SNP Wald ratio only; the instrument had F = 9. Heterogeneity, weighted-median, MR-Egger, and leave-one-out analyses were not applicable. |

**Note.** F < 10 was treated as weak-instrument evidence. MEHHP contained only one instrument; therefore, multi-SNP sensitivity analyses were not applicable.

**B. Primary MR estimates and sensitivity analyses**

| **Exposure** | **Outcome** | **Source** | **n SNPs** | **Primary estimate beta** | **Primary estimate SE** | **Primary estimate P** | **Cochran Q P** | **Weighted-median beta [95% CI]** | **MR-Egger slope P / intercept P** | **Leave-one-out interpretation** |
| --- | --- | --- | --- | --- | --- | --- | --- | --- | --- | --- |
| MECPP | NAFLD | FinnGen | 4 | -0.049 | 0.258 | 0.848 | 0.993 | -0.088 [-0.134, 0.153] | 0.909 / 0.549 | All leave-one-out estimates remained non-significant. |
| MEHHP | NAFLD | FinnGen | 1 | -0.176 | 1.038 | 0.865 | NA | NA | NA | Not applicable because only one SNP was available; the primary estimate was obtained using the Wald ratio. |
| MECPP | Fibrosis/cirrhosis | FinnGen | 4 | -0.294 | 0.296 | 0.321 | 0.433 | -0.031 [-1.388, 0.353] | 0.676 / 0.359 | All leave-one-out estimates remained non-significant. |
| MEHHP | Fibrosis/cirrhosis | FinnGen | 1 | -0.560 | 1.191 | 0.638 | NA | NA | NA | Not applicable because only one SNP was available; the primary estimate was obtained using the Wald ratio. |
| MECPP | Toxic liver disease | FinnGen | 4 | -1.201 | 0.667 | 0.072 | 0.500 | -0.314 [-3.046, -0.314] | 0.789 / 0.561 | The overall estimate was non-significant; omission of rs4918798 yielded a nominally significant inverse estimate (beta = -2.228, P = 0.023), indicating instability. |
| MEHHP | Toxic liver disease | FinnGen | 1 | 0.651 | 2.693 | 0.809 | NA | NA | NA | Not applicable because only one SNP was available; the primary estimate was obtained using the Wald ratio. |
| MECPP | HCC | FinnGen | 4 | 0.230 | 0.496 | 0.643 | 0.545 | 0.040 [-1.390, 1.220] | 0.157 / 0.172 | All leave-one-out estimates remained non-significant. |
| MEHHP | HCC | FinnGen | 1 | 0.678 | 2.001 | 0.735 | NA | NA | NA | Not applicable because only one SNP was available; the primary estimate was obtained using the Wald ratio. |
| MECPP | ALT | UKB | 4 | -0.023 | 0.024 | 0.332 | 0.112 | 0.017 [-0.144, 0.017] | 0.966 / 0.818 | The overall estimate was non-significant; omission of rs4918798 yielded a nominally significant inverse estimate (beta = -0.081, P = 0.029), indicating instability. |
| MECPP | AST | UKB | 4 | -0.077 | 0.024 | 0.002 | 0.717 | -0.056 [-0.128, -0.056] | 0.283 / 0.528 | The inverse association remained nominally significant after removal of each SNP (all P < 0.01). |
| MECPP | ALP | UKB | 4 | 0.088 | 0.025 | 4.02e-4 | 3.99e-8 | 0.136 [-0.355, 0.201] | 0.019 / 0.026 | The association was not robust: omission of rs4918798 yielded beta = 0.018 and P = 0.635. Substantial heterogeneity, an MR-Egger intercept signal, and a weighted-median confidence interval crossing zero further limited interpretation. |
| MECPP | GGT | UKB | 4 | -0.019 | 0.024 | 0.417 | 0.117 | 0.028 [-0.097, 0.028] | 0.887 / 0.690 | The overall estimate was non-significant; omission of rs4918798 yielded a nominally significant inverse estimate (beta = -0.088, P = 0.017), indicating instability. |
| MECPP | Total bilirubin | UKB | 4 | 0.004 | 0.025 | 0.860 | 0.004 | 0.036 [-0.263, 0.048] | 0.022 / 0.018 | All leave-one-out estimates remained non-significant; however, heterogeneity and an MR-Egger intercept signal limited interpretation. |
| MEHHP | ALT | UKB | 1 | 0.063 | 0.093 | 0.496 | NA | NA | NA | Not applicable because only one SNP was available; the primary estimate was obtained using the Wald ratio. |
| MEHHP | AST | UKB | 1 | -0.163 | 0.095 | 0.085 | NA | NA | NA | Not applicable because only one SNP was available; the primary estimate was obtained using the Wald ratio. |
| MEHHP | ALP | UKB | 1 | 0.404 | 0.097 | 2.91e-5 | NA | NA | NA | Not applicable because only one SNP was available; the primary estimate was obtained using the Wald ratio. |
| MEHHP | GGT | UKB | 1 | 0.052 | 0.092 | 0.576 | NA | NA | NA | Not applicable because only one SNP was available; the primary estimate was obtained using the Wald ratio. |
| MEHHP | Total bilirubin | UKB | 1 | 0.134 | 0.095 | 0.160 | NA | NA | NA | Not applicable because only one SNP was available; the primary estimate was obtained using the Wald ratio. |

**Note.** Primary estimates were calculated using first-order inverse-variance weighting for MECPP and the Wald ratio for MEHHP. Multi-SNP sensitivity analyses, including Cochran Q, weighted-median, MR-Egger, and leave-one-out analyses, were not applicable to MEHHP because only one instrument was available. Weighted-median confidence intervals were calculated from the empirical 2.5th and 97.5th percentiles of 5,000 SNP-resampling bootstrap replicates. Because only four MECPP instruments were available, the bootstrap distributions were discrete, and a confidence-limit value could coincide with the point estimate. Weighted-median and MR-Egger results should therefore be interpreted as exploratory sensitivity analyses rather than precise or independent causal estimates. Nominal significance refers to P < 0.05 without correction for multiple comparisons.

**Abbreviations:** ALP, alkaline phosphatase; ALT, alanine aminotransferase; AST, aspartate aminotransferase; GGT, gamma-glutamyl transferase; HCC, hepatocellular carcinoma; IVW, inverse-variance weighted; LOO, leave-one-out; MR, Mendelian randomization; NA, not applicable; NAFLD, non-alcoholic fatty liver disease; UKB, UK Biobank.
